# Supplementary material for: The role of SPP1 in evaluating the prognosis, immune infiltration, and drug sensitivity of hepatocellular carcinoma
Source: PLoS One. 2026 Apr 22;21(4):e0347842. doi: 10.1371/journal.pone.0347842 (PMC13102187; doi:10.1371/journal.pone.0347842)
Supplement: S1 Table — (DOCX) [file pone.0347842.s001.docx]

Supplementary Material 1. qRT-PCR Protocol and Primer Sequences.

| SPP1 Forward | 5'-TGAAACGAGTCAGCTGGATG-3' |
| --- | --- |
| SPP1 Reverse | 5'-CAGGTCTGCGAAACTTCTTA-3' |
| GAPDH Forward | 5'-GGAGCGAGATCCCTCCAAAAT-3' |
| GAPDH Reverse | 5'-GGCTGTTGTCATACTTCTCATGG-3' |
