# Supplementary material for: The role of SPP1 in evaluating the prognosis, immune infiltration, and drug sensitivity of hepatocellular carcinoma
Source: PLoS One. 2026 Apr 22;21(4):e0347842. doi: 10.1371/journal.pone.0347842 (PMC13102187; doi:10.1371/journal.pone.0347842)
Supplement: S2 Table — (DOCX) [file pone.0347842.s002.docx]

| Drug | Low SPP1 IC50 | High SPP1 IC50 | P-value |
| --- | --- | --- | --- |
| 17−AAG | 1.83 | -0.89 | 1e-06 |
| A−770041 | 3.56 | 2.52 | 0.00042 |
| AG−014699 | 3.78 | 3.31 | 0.00094 |
| BI−2536 | -1.52 | -1.82 | 0.0007 |
| Bortezomib | -4.78 | -5.84 | 3e-06 |
| CGP−60474 | -1.25 | -1.98 | 1.5e-05 |
| CGP−082996 | 3.11 | 2.39 | 5.2e-05 |
| Dasatinib | 3.74 | 1.24 | 0.00037 |
| FR−180204 | 5.46 | 5.12 | 5.2e-05 |
| GW843682X | 0.18 | -1.78 | 0.00092 |
| Imatinib | 3.10 | 2.56 | 3.1e-11 |
| JW−7−52−1 | -1.25 | -1.85 | 0.00075 |
| KIN001−102 | 3.11 | 2.75 | 0.00073 |
| LY317615 | 4.41 | 3.52 | 4.7e-11 |
| Mitomycin C | 0.86 | -0.78 | 0.00011 |
| MS−275 | 1.48 | 0.95 | 5.7e-05 |
| Paclitaxel | -2.36 | -3.35 | 2.4e-10 |
| Pyrimethamine | 5.41 | 4.68 | 2.4e-09 |
| Sorafenib | 2.78 | 2.45 | 3.5e-05 |
| S−Trityl−L−cysteine | 1.99 | 1.56 | 5.8e-08 |
| Sunitinib | 4.15 | 2.24 | 5.1e-13 |
| TAE684 | 1.89 | 1.17 | 0.00015 |
| THZ−2−49 | 3.91 | 2.39 | 1e-05 |
| VX−680 | 2.56 | 1.99 | 0.00029 |
| WZ−1−84 | 4.35 | 4.14 | 0.00053 |
| (5Z)−7−Oxozeaenol | 0.625 | 1.26 | 1.2e-05 |
| BX−795 | 1.47 | 1.95 | 0.00022 |
| Camptothecin | -5.23 | -3.84 | 1.5e-09 |
| CEP−701 | -1.25 | -0.10 | 7.6e-05 |
| CI−1040 | 2.67 | 3.08 | 0.00033 |
| CX−5461 | 2.89 | 3.68 | 2.5e-06 |
| EHT 1864 | 3.28 | 3.61 | 1e-05 |
| Elesclomol | -3.72 | -2.75 | 6e-06 |
| FH535 | 1.54 | 2.07 | 1.2e-05 |
| FTI−277 | 1.82 | 2.14 | 3.6e-07 |
| GDC0449 | 3.87 | 4.48 | 6.7e-08 |
| GSK429286A | 4.72 | 5.10 | 0.00031 |
| JNJ−26854165 | 2.27 | 2.85 | 4.3e-05 |
| KIN001−135 | 3.67 | 3.87 | 0.00036 |
| KU−55933 | 3.47 | 3.95 | 6.2e-06 |
| Methotrexate | -1.05 | -0.25 | 0.00089 |
| NSC−207895 | 3.61 | 4.15 | 7.6e-05 |
| NU−7441 | 2.00 | 2.56 | 1e-07 |
| OSI−027 | -0.54 | 1.25 | 1.3e-09 |
| PI−103 | 0.49 | 1.25 | 6.1e-06 |
| piperlongumine | 1.68 | 2.11 | 0.00049 |
| QL−X−138 | 0.97 | 1.61 | 0.00022 |
| QL−XI−92 | 3.52 | 4.07 | 0.0004 |
| QL−XII−47 | 0.55 | 1.28 | 7.8e-05 |
| SN−38 | -6.14 | -4.12 | 3.7e-08 |
| SNX−2112 | -2.14 | -0.84 | 0.00014 |
| Temsirolimus | -3.14 | -1.98 | 0.0006 |
| THZ−2−102−1 | -2.56 | -1.54 | 0.00011 |
| Trametinib | -1.25 | 1.06 | 0.00082 |
| TW 37 | -1.00 | -0.48 | 4.4e-05 |
| Vorinostat | 0.30 | 1.52 | 0.00012 |
| VX−702 | 2.51 | 3.61 | 6.2e-06 |
| XMD13−2 | 3.21 | 3.74 | 0.0002 |
| YM155 | 5.04 | 6.14 | 0.00013 |
| ZSTK474 | 0.85 | 1.34 | 0.00026 |
