# Supplementary material for: The role of SPP1 in evaluating the prognosis, immune infiltration, and drug sensitivity of hepatocellular carcinoma
Source: PLoS One. 2026 Apr 22;21(4):e0347842. doi: 10.1371/journal.pone.0347842 (PMC13102187; doi:10.1371/journal.pone.0347842)
Supplement: S1 Fig — (PDF) [file pone.0347842.s003.pdf]

Fig 7B

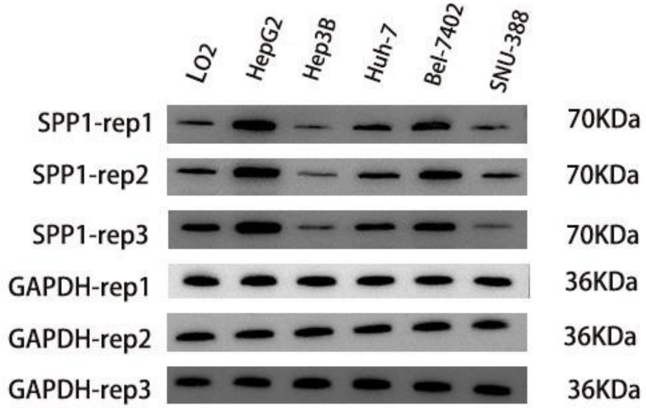

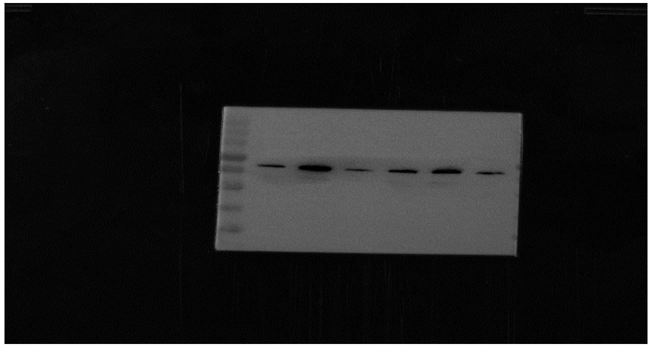

SPP1-rep1

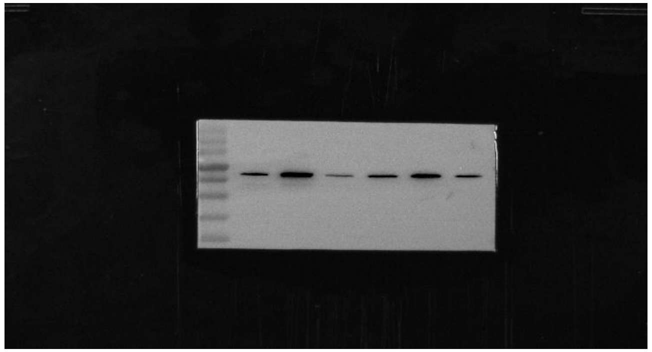

SPP1-rep2

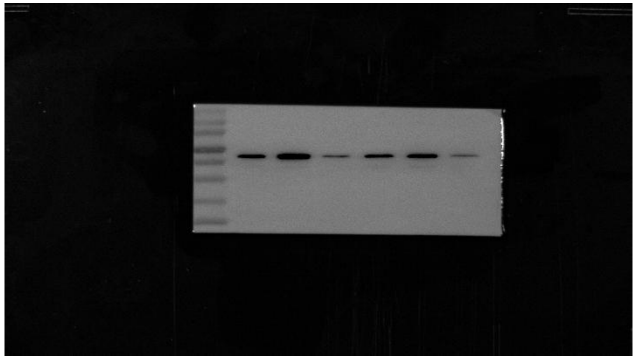

SPP1-rep3

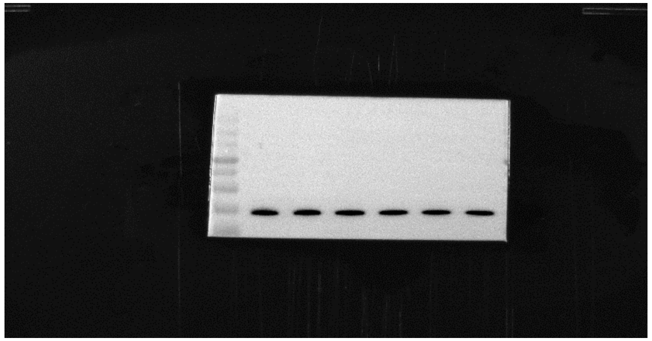

GAPDH-rep1

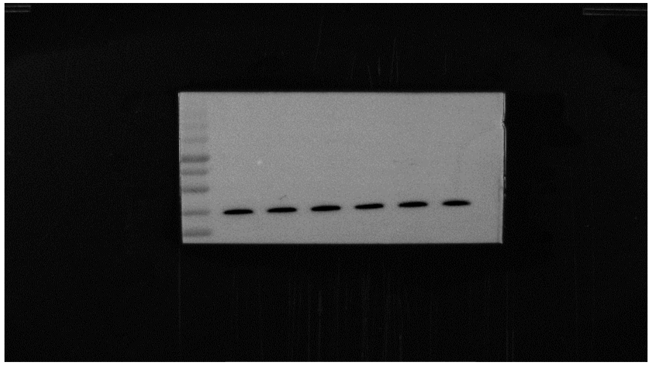

GAPDH-rep2

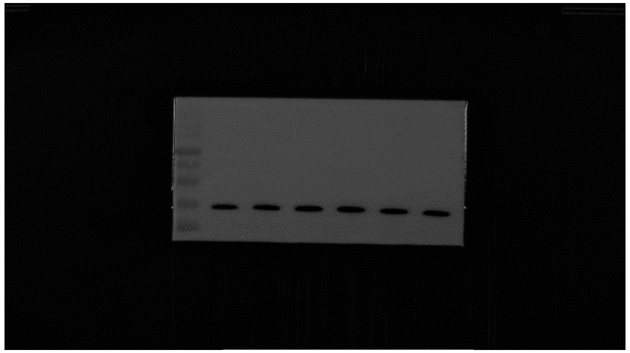

GAPDH-rep3

Fig 7E

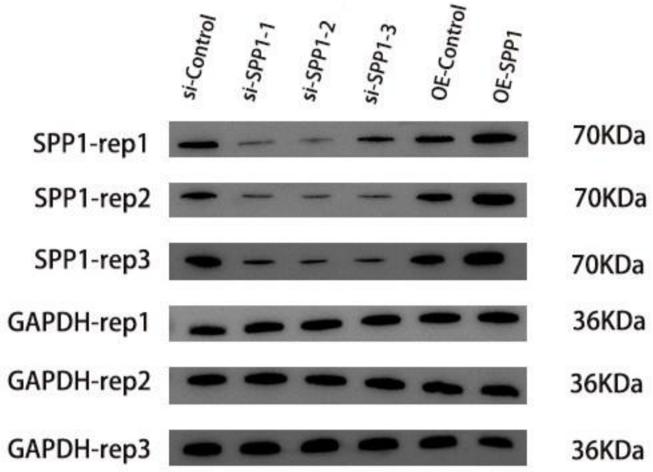

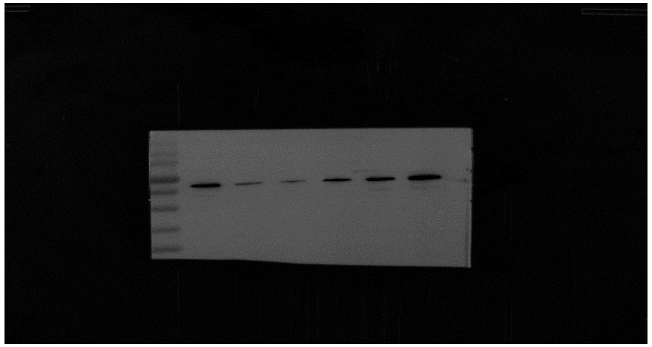

SPP1-rep1

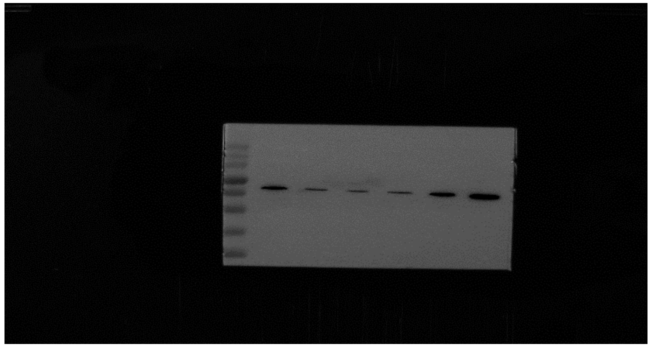

SPP1-rep2

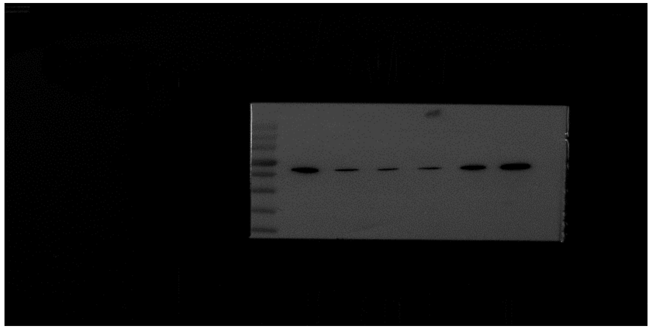

SPP1-rep3

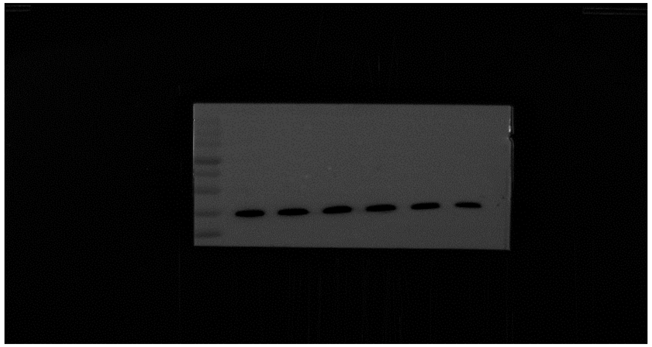

GAPDH-rep1

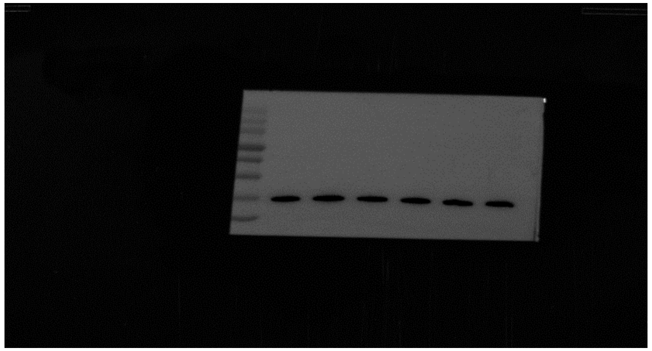

GAPDH-rep2

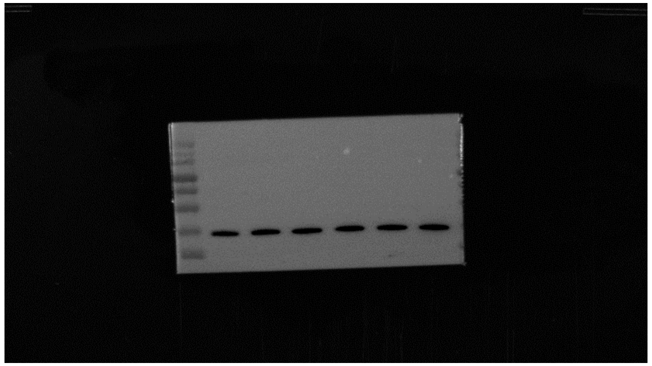

GAPDH-rep3
