# Supplementary material for: The role of SPP1 in evaluating the prognosis, immune infiltration, and drug sensitivity of hepatocellular carcinoma
Source: PLoS One. 2026 Apr 22;21(4):e0347842. doi: 10.1371/journal.pone.0347842 (PMC13102187; doi:10.1371/journal.pone.0347842)
Supplement: Attachment — Submitted filename: Response to Reviewers.docx [file pone.0347842.s005.docx]

Journal Requirements

1. Please ensure that your manuscript meets PLOS ONE's style requirements, including those for file naming.

Response: I have made further revisions according to the style of the journal, as detailed in my resubmitted manuscript. If there are any incorrect modifications, please raise them and I will continue to make them carefully.

2. Thank you for stating the following financial disclosure: “the Science and Technology Program of the Joint Fund of Scientific Research for the Public Hospitals of Inner Mongolia Academy of Medical Sciences (Grant No. 2024GLLH0862).” Please state what role the funders took in the study. If the funders had no role, please state: "The funders had no role in study design, data collection and analysis, decision to publish, or preparation of the manuscript." If this statement is not correct you must amend it as needed. Please include this amended Role of Funder statement in your cover letter; we will change the online submission form on your behalf.

3. Please note that funding information should not appear in any section or other areas of your manuscript. We will only publish funding information present in the Funding Statement section of the online submission form. Please remove any funding-related text from the manuscript.

Response: I have made revisions to the relevant suggestions regarding the second and third points, as detailed in my resubmitted cover letter. In addition, I have removed the relevant content from the resubmitted manuscript.

4. Please provide a complete Data Availability Statement in the submission form, ensuring you include all necessary access information or a reason for why you are unable to make your data freely accessible. If your research concerns only data provided within your submission, please write "All data are in the manuscript and/or supporting information files" as your Data Availability Statement.

Response: I have added the sentence 'All data is in the manuscript and/or supporting information files' to the' Data Availability Statement '.

5. PLOS ONE now requires that authors provide the original uncropped and unadjusted images underlying all blot or gel results reported in a submission’s figures or Supporting Information files. In your cover letter, please note whether your blot/gel image data are in Supporting Information or posted at a public data repository, provide the repository URL if relevant, and provide specific details as to which raw blot/gel images.

Response: I have placed my blot image data in the supporting information and agree to publish the above data.

6. Please include captions for your Supporting Information files at the end of your manuscript, and update any in-text citations to match accordingly.

Response: I have made revisions to it, please refer to my resubmitted manuscript for details.

7. If the reviewer comments include a recommendation to cite specific previously published works, please review and evaluate these publications to determine whether they are relevant and should be cited. There is no requirement to cite these works unless the editor has indicated otherwise.

Response: The reviewer did not make any relevant requests.

8. Please review your reference list to ensure that it is complete and correct. If you have cited papers that have been retracted, please include the rationale for doing so in the manuscript text, or remove these references and replace them with relevant current references. Any changes to the reference list should be mentioned in the rebuttal letter that accompanies your revised manuscript. If you need to cite a retracted article, indicate the article’s retracted status in the References list and also include a citation and full reference for the retraction notice.

Response: I have reviewed the paper I cited again and there are no references that have been retracted.

Reviewer 1:

1. The research was confined to HepG2 cells. To improve the study's strength and reliability, it is advisable to incorporate a broader spectrum of hepatocellular carcinoma (HCC) cell lines. Furthermore, expanding the investigation to include in vivo models would be advantageous.

Response: You are absolutely right, this study is indeed limited to HepG2 cells. The application of more hepatocellular carcinoma cell lines will be more convincing. However, despite funding support, this study has limited resources and can only use HepG2 cells for experiments. If there is an opportunity in the future, more cell lines will be used for research to enhance persuasiveness.

2. The mechanistic depth is insufficient; although enrichment analyses suggest the involvement of PI3K-Akt and immune-related pathways, no direct mechanistic experiments, such as pathway inhibition or Western blotting for downstream signaling proteins, were conducted to confirm pathway activation.

Response: Thank you for your careful review and valuable feedback on this article. The issues you pointed out, such as insufficient mechanism depth and the need for direct mechanism experimental verification, are very relevant and we fully agree with them. But equally, our funds are very limited, and we will further research in this direction once we have sufficient funds in the future.

3. The interpretation of immune infiltration is speculative, as conclusions regarding macrophage polarization and CD8+ T-cell suppression lack direct experimental validation through methods such as flow cytometry or immunohistochemistry.

Response: What you said is correct, I have made modifications based on your suggestions. If there is anything else that you are not satisfied with, please raise it and I will continue to make serious revisions.

Page 15:

Before revise: Consequently, we investigated whether SPP1 is linked to tumor immunity in HCC.

After revise: Therefore, we investigated the relationship between SPP1 expression and immune-related features in HCC.

Page 15:

Before revise: According to our research, SPP1 serves a crucial role in the infiltration of neutrophils, macrophages, myeloid dendritic cells, and B cells.

After revise: In our analyses, SPP1 expression was associated with transcriptional signatures related to neutrophils, macrophages, myeloid dendritic cells, and B cells.

Page 15:

Before revise: Macrophages, which originate from monocytes, play a crucial role in both specific and non-specific immunity, enabling the host to maintain the internal balance of tissues [34, 35]. The phenotypic diversity of macrophages results in variations in their functions when they encounter tumors, and even opposite effects can occur.

Before revise: Macrophages, derived from monocytes, are key effectors of innate and adaptive immunity and exhibit phenotypic plasticity in tumors; different macrophage phenotypes can exert distinct and sometimes opposing effects on tumor biology [34, 35].

4. Statistical clarity is required; the DEG threshold is reported as “logFC < 1,” which appears to be a typographical error and should be |logFC| > 1.

Response: Indeed, this was my mistake. I have already made modifications to it.

Page 6:

Before revise: Two groups of differentially expressed genes (DEGs) were screened using the threshold criteria of logFC < 1, and FDR < .05.

After revise: Two groups of differentially expressed genes (DEGs) were screened using the threshold criteria of |logFC| > 1 and FDR < .05.

5. Several typographical and spacing errors are present throughout the manuscript and should be corrected for clarity.

Response: Thank you for your reminder. The editor of the journal has asked me to further modify the format according to the journal's requirements.

6. Inconsistencies are noted in some figure legends, such as EXOSC10 being mentioned in Figure 1C instead of SPP1.

Response: This is indeed my mistake. I have already made modifications to it.

Before revise: (C) EXOSC10 was significantly elevated in 374 HCC samples compared to the corresponding adjacent liver tissue. In databases GSE45436, GSE54236, GSE121248, GSE76427, GSE64041, and GSE60502, SPP1 expression is higher in HCC samples compared to normal samples.

After revise: (C) SPP1 was significantly elevated in 374 HCC samples compared to the corresponding adjacent liver tissue. In databases GSE45436, GSE54236, GSE121248, GSE76427, GSE64041, and GSE60502, SPP1 expression is higher in HCC samples compared to normal samples.

7. The Discussion section could be more concise and better focused on the study’s novel contributions.

Response: I have made modifications based on your feedback. If further modifications are needed, please let me know and I will continue to make them.

Page 17

Before revise: This study used comprehensive bioinformatics to investigate the connection between SPP1 and HCC. While chemotherapy, targeted therapy, and immune checkpoint inhibitors represent important treatment options for advanced HCC, not all patients respond effectively. Therefore, identifying treatment targets helps to select the most suitable drugs, thereby better implementing precision treatment and enabling patients to achieve higher survival. As a result, SPP1 may be targeted for HCC as a result of this study's research, offering a novel approach to the treatment of HCC patients. This article still has many of restrictions, though. To begin with, in terms of experiments, all we did was carry out a basic confirmation of SPP1's involvement in HCC. More research is required to determine how SPP1 influences the onset and progression of HCC. Second, even though SPP1 and tumor immune infiltration were shown to be significantly correlated in our study, the exact mechanism behind this finding is still unknown and needs to be confirmed by more research.

After revise: In this study, we provide a multi-layered characterization of SPP1 in hepatocellular carcinoma (HCC). Across TCGA, six GEO datasets, and HPA protein data, SPP1 was consistently upregulated at the mRNA and protein levels. High SPP1 expression was associated with poorer overall and progression‑free survival and with more advanced clinicopathologic features. In vitro, SPP1 knockdown reduced HCC cell proliferation, migration, and invasion and increased apoptosis, while SPP1 overexpression had opposite effects.

Page 14

Before revise: SPP1, located on chromosome 4q13 and consisting of seven exons and six introns, encodes a 294-amino-acid secreted phosphoprotein [27]. In humans, three domains of SPP1 bind to integrins: the RGD domain, the SVVYGLR domain, and the ELVTDFPTDLPAT domain [28]. Among them, the most well-known ones are the RGD domain and the SVVYGLR domain. SPP1 promotes tumor development and progression by binding to CD44 and integrin receptors. Through interaction with CD44 and αvβ3 integrin, SPP1 activates the PI3K/Akt/HIF‑1α and PI3K/Akt/NF‑κB signaling pathways, thereby enhancing tumor angiogenesis and epithelial‑mesenchymal transition (EMT), respectively [29,30]. Additionally, SPP1 binding to αvβ3 integrin triggers the JNK pathway, leading to upregulation of MMP2 and MMP9 expression and facilitating tumor invasion [30]. Furthermore, SPP1 attaches itself to α9β1 integrin, initiating the p38 and ERK signaling pathways and increasing the production of COX-2, a protein that promotes the migration of tumor cells [31]. Although SPP1 has been linked to multiple malignancies [32], its prognostic significance and underlying mechanisms in HCC remain incompletely understood.

After revise: SPP1 encodes a 294–amino-acid secreted phosphoprotein located on chromosome 4q13 [27]. SPP1 contains integrin-binding domains including RGD and SVVYGLR [28]. The RGD and SVVYGLR domains are the best characterized. SPP1 promotes tumor progression via interactions with CD44 and integrins. Through interaction with CD44 and αvβ3 integrin, SPP1 activates the PI3K/Akt/HIF‑1α and PI3K/Akt/NF‑κB signaling pathways, thereby enhancing tumor angiogenesis and epithelial‑mesenchymal transition (EMT), respectively [29,30]. Additionally, SPP1 binding to αvβ3 integrin triggers the JNK pathway, leading to upregulation of MMP2 and MMP9 expression and facilitating tumor invasion [30]. Interaction with α9β1 activates p38/ERK and upregulates COX 2, enhancing tumor cell migration [31]. Despite associations with various cancers, SPP1’s prognostic role and mechanisms in HCC are not fully defined [32].

Page 14

Before revise: We also used six independent GEO databases (GSE45436, GSE54236, GSE121248, GSE76427, GSE64041, and GSE60502) for validation. Furthermore, in HCC patients, higher SPP1 expression is associated with worse OS and PFS. Meanwhile, we verified that SPP1 may function as a stand-alone risk factor for assessing the prognosis of HCC patients and is connected to the histological grade, pathological grade, and T stage of tumors.

After revise: We validated SPP1 upregulation using six independent GEO cohorts. Clinically, high SPP1 correlates with poorer OS and PFS. Multivariate analysis indicates SPP1 is an independent prognostic factor and associates with higher histologic grade and T stage.

8. Ethical approval and cell line authentication statements are not clearly described.

Response: Thank you for your reminder, I did indeed miss this point. I have added this section to the manuscript.

Page 5: The data used in this study were all from public databases, and the cell lines used were commercially purchased. Therefore, this study did not require specific ethical approval.

Reviewer 2:

1. Introduction: Referencing needs editing there should be comma in between 23 same goes with 56,911,1213 throughout the introduction written part.

Response: I do have some shortcomings in terms of references, and I have revised them accordingly.

2. “In 2020, hepatocellular carcinoma (HCC) had a global population of 910,000 cases and 830,000 deaths, which is a common malignant tumor.1 In terms of global malignant tumors, the number of new cases and deaths ranks sixth and fourth, respectively.1” These two sentences can be merged into one effective sentence.

Response: These two sentences are indeed a bit redundant, I have merged them together.

Page 3

Before revise: In 2020, hepatocellular carcinoma (HCC) had a global population of 910,000 cases and 830,000 deaths, which is a common malignant tumor. In terms of global malignant tumors, the number of new cases and deaths ranks sixth and fourth, respectively.

After revise: In 2020, hepatocellular carcinoma (HCC), a common malignant tumor, had a global burden of approximately 910,000 new cases and 830,000 deaths, ranking sixth and fourth, respectively, in terms of incidence and mortality worldwide.

3.“Initially, the mRNA expression level of SPP1 was assessed using publicly available databases,……………………………..”. After this author have mentioned that “We also investigated the association between SPP1 expression and immune………….”. This paragraph can be reframed meaning since authors are trying to say that what they have done in this research work.

Response: Thank you for your reminder, I have made further modifications.

Page 4

Before revise: Initially, the mRNA expression level of SPP1 was assessed using publicly available databases, including The Cancer Genome Atlas (TCGA), Gene Expression Omnibus (GEO), and the Human Protein Atlas (HPA). Subsequently, the prognostic value of SPP1 in HCC was further validated with TCGA survival data. We also investigated the association between SPP1 expression and immune cell infiltration within the tumor microenvironment. In addition, the potential of SPP1 as a biomarker for predicting drug sensitivity in HCC was systematically evaluated. To experimentally verify its role, SPP1 was knocked out in HCC cell lines, and its expression was examined in vitro. Collectively, this study elucidates the important functions of SPP1 in HCC progression and therapy.

After revise: This study aims to comprehensively elucidate the role of SPP1 in HCC progression and therapy. To achieve this, we performed a multi-faceted study. First, we assessed the mRNA and protein expression levels of SPP1 in HCC using publicly available databases, including The Cancer Genome Atlas (TCGA), Gene Expression Omnibus (GEO), and the Human Protein Atlas (HPA). Subsequently, the prognostic value of SPP1 in HCC was further validated with TCGA survival data. We also investigated the association between SPP1 expression and immune cell infiltration within the tumor microenvironment. In addition, the potential of SPP1 as a biomarker for predicting drug sensitivity in HCC was systematically evaluated. To experimentally verify its functional role, SPP1 was knocked down or overexpressed in HCC cell lines, and its effects on proliferation, migration, invasion, and apoptosis were examined in vitro.

4. In vitro should be in italics.

Response: I have edited it all in italics.

5. Materials and Methods: This section is well-written and shows great attention to detail. However, authors have not provided information regarding the expression of genes in hepatocellular carcinoma (HCC) cell lines; rather, they have solely addressed the cultivation of these cells.

Response: Thank you for your revision suggestion. I have added the corresponding description in the paper.

To identify a representative HCC cell line for functional assays, the basal expression levels of SPP1 were first evaluated in a panel of human HCC cell lines and the normal human liver cell line. Total RNA was extracted from these cell lines using TRIzol reagent (Invitrogen) according to the manufacturer's protocol. cDNA was synthesized from 1-2 μg of total RNA using a PrimeScript RT reagent kit (Takara). Quantitative real-time PCR (qRT-PCR) was performed with SYBR Green PCR master mix (Applied Biosystems) on a QuantStudio 3 Real-Time PCR System.

6. Figure legends could be little bit descriptive for example Fig 1, there is no description in the legends about 1D-I. Moreover, for Fig1J authors can mentioned instead of “HPA database, the expression of SPP1 protein is elevated in HCC tissues compared to non-HCC tissues” as shown in left circle or right circle. It is not quite clear for the reader.

Response: I have modified the legend of my Figure 1. If there is any dissatisfaction, please raise it. I will continue to make serious revisions.

Page 19

Fig 1. The expression of SPP1 in normal and liver cancer tissues. (A) SPP1 gene expression in 33 tumor tissues in TIMER database. (B) Compared with normal samples, SPP1 expression is higher in HCC samples. (C) SPP1 was significantly elevated in 374 HCC samples compared to the corresponding adjacent liver tissue. (D-I) In databases GSE45436, GSE54236, GSE121248, GSE76427, GSE64041, and GSE60502, SPP1 expression is higher in HCC samples compared to normal samples. (J) In the HPA database, the expression of SPP1 protein is elevated in HCC tissues (right circle) compared to non HCC tissues (left circle).

7. Regarding Figures 5 and 6, if the authors are presenting IC50 values, it would be more effective to display them in a table format, as the graphical representation does not add significant value. Alternatively, they could create a representative graph that differentiates between low and high IC50 values and then compile the data in a table, categorizing the drugs into low and high IC50 groups.

Response: I have added additional forms and materials as per your request.

8. In Fig 7C is it SNU-387 or 388 because WB showed as 388.

Response: Thank you for your reminder. My HCC cell line is SUN-387 and has been corrected.

9. “The KEGG enrichment pathway primarily included neuroactive ligand-receptor interaction,
cytoskeleton in muscle cells, cytokine-cytokine receptor interaction, and the PI3K-Akt signaling pathway, among others (Figure 3D).” This statement is not very clear in terms of figure representation.

Response: Indeed, there are issues with the wording of the image, and I have made further modifications according to your request. If you are not satisfied, please let us know. I will continue to make serious revisions.

Before revise: (D) The KEGG pathway of differentially expressed genes in HCC.

After revise: (D) Bar plot of KEGG enrichment analysisr esults.

10. “Results revealed that high SPP1 expression was correlated with significantly higher immune and ESTIMATE scores compared to the low-expression group (Figure 4A).” Which results authors are trying to refer it in here?

Response: This is indeed my mistake. I have already made modifications to it.

Page 12

Before revise: Results revealed that high SPP1 expression was correlated with significantly higher immune and ESTIMATE scores compared to the low-expression group (Fig 4A).

After revise: There was a statistically significant difference in immune scores and ESTIMATE scores between the low SPP1 group and the high SPP1 group (Fig 4A).

11. It can be re-written as “To validate the findings from the in-silico gene analysis, we examined the expression of SPP1 in HCC cell lines, either through transient transfection or stable integration”.

Response: Thank you for your reminder. Your expression is better, I have made revisions.

To validate bioinformatics findings, SPP1 expression was examined in HCC cell lines.

To validate the findings from the in silico analysis, we modulated SPP1 expression in HCC cell lines using transient transfection.

12. However, the authors did not specify which method was used for the expression of SPP1 in HCC cell lines, either in the main text or in the figure legends. This information is crucial, as the relevance of the data obtained depends significantly on whether SPP1 expression was transient or stably integrated.

Response: Thank you for your reminder. I have added the above content to the Materials and Methods section.

Cell transfection

SPP1 expression was transiently manipulated in HCC cell lines using siRNA-mediated knockdown and plasmid-mediated overexpression. For knockdown experiments, cells were transfected with SPP1-specific siRNAs (si-SPP1-1, si-SPP1-2, and si-SPP1-3) or a negative control siRNA. For overexpression, cells were transfected with an SPP1 expression plasmid (OE-SPP1) or an empty vector control. Transfections were performed usingLipofectamine 3000 according to the manufacturer’s instructions.

13. This can be introduced into the introduction “SPP1, located on chromosome 4q13 and consisting of seven exons and six introns, encodes a 294-amino-acid secreted phosphoprotein. In humans, three domains of SPP1 bind to integrins: the RGD domain, the SVVYGLR domain, and the ELVTDFPTDLPAT domain. Among them, the most well-known ones are the RGD domain and the SVVYGLR domain. SPP1 promotes tumor development and progression by binding to CD44 and integrin receptors.” Because these findings regarding SPP1 do not contribute meaningfully to the discussion, as the authors have not conducted any biochemical or biophysical assays to emphasize the significance of these domains.

Response: Thanks for the reminder. I have added the relevant content to the introduction section.

After revise: SPP1 encodes a 294–amino-acid secreted phosphoprotein located on chromosome 4q13. SPP1 contains integrin-binding domains including RGD and SVVYGLR. The RGD and SVVYGLR domains are the best characterized. SPP1 promotes tumor progression via interactions with CD44 and integrins.

14. “In this work, we employed bioinformatics techniques to assess the function of SPP1 in relation to the expression, prognosis, and therapeutic sensitivity of HCC patients, and we verified our findings through specific experiments.” Authors needs to mention what specific experiments.

Response: Thank you for the reminder, I have made the necessary modifications.

Before revise: In this work, we employed bioinformatics techniques to assess the function of SPP1 in relation to the expression, prognosis, and therapeutic sensitivity of HCC patients, and we verified our findings through specific experiments.

After revise: In this work, we employed bioinformatics techniques to assess the role of SPP1 in the expression, prognosis, and therapeutic sensitivity of HCC patients, and validated our findings through qRT-PCR, Western blotting, and functional assays including CCK-8, colony formation, wound healing, Transwell invasion, and TUNEL assays.

15. “Meanwhile, we verified that SPP1 may function as a stand-alone risk factor for assessing the prognosis of HCC patients and is connected to the histological grade, pathological grade, and T stage of tumors.” Authors should rewrite this like In addition to above mentioned findings, we have verified……….

Response: Thank you for your reminder. Your review suggestions are the same as those of the first reviewer, and I have made revisions in the discussion section.

Before revise: Meanwhile, we verified that SPP1 may function as a stand-alone risk factor for assessing the prognosis of HCC patients and is connected to the histological grade, pathological grade, and T stage of tumors.

After revise: Multivariate analysis indicates SPP1 is an independent prognostic factor and associates with higher histologic grade and T stage.

16. “Consequently, we investigated whether SPP1 is linked to tumor immunity in HCC.” This can be expressed in a more scientific manner, emphasizing author’s unique contributions rationale behind it rather than simply paralleling previous experiments conducted by other groups.

Response: Thank you for your reminder. Your review suggestions are the same as those of the first reviewer, and I have made revisions in the discussion section.

Before revise: Consequently, we investigated whether SPP1 is linked to tumor immunity in HCC.

After revise: Therefore, we investigated the relationship between SPP1 expression and immune-related features in HCC.

17. I appreciated the authors' honesty in expressing this “This article still has many of restrictions, though. To begin with, in terms of experiments, all we did was carry out a basic confirmation of SPP1's involvement in HCC. More research is required to determine how SPP1 influences the onset and progression of HCC. Second, even though SPP1 and tumor immune infiltration were shown to be significantly correlated in our study, the exact mechanism behind this finding is still unknown and needs to be confirmed by more research.” However, authors can write this in a more scientific manner. For example “This article, however, presents several limitations. Firstly, our experimental approach was limited to a basic validation of the involvement of SPP1 in hepatocellular carcinoma (HCC). Further investigation is necessary to elucidate the role of SPP1 in the initiation and progression of HCC. Secondly, while our study identified a significant correlation between SPP1 and tumor immune infiltration, the precise mechanisms underlying this relationship remain unclear and warrant additional research for confirmation”.

Response: Thank you for your reminder. I have made further modifications according to your request.

Before revise: In summary, our study supports the potential of SPP1 as a prognostic biomarker and therapeutic target in HCC. However, several limitations should be acknowledged. Firstly, the expression and prognostic analyses of SPP1 rely on retrospective data from public databases. Although sample sizes are substantial, prospective clinical studies are required to further validate its prognostic utility. Secondly, the analysis of immune cell infiltration utilizes computational methods such as CIBERSORT. Although the algorithm has been validated, there is a lack of direct verification of immune cell composition through experimental methods such as flow cytometry and immunohistochemistry. Thirdly, in vitro experiments only utilize the HepG2 cell line. The effects of SPP1 on cell proliferation, migration, and invasion should be validated in more HCC cell lines and animal models. Finally, drug sensitivity prediction is based on cell line data from the GDSC database, which may differ from the actual therapeutic effect in clinical patients. It is necessary to validate these results in combination with clinical samples.

After revise: This article, however, presents several limitations. Firstly, our experimental approach was limited to a basic validation of the involvement of SPP1 in hepatocellular carcinoma (HCC). Further investigation is necessary to elucidate the role of SPP1 in the initiation and progression of HCC. Secondly, while our study identified a significant correlation between SPP1 and tumor immune infiltration, the precise mechanisms underlying this relationship remain unclear and warrant additional research for confirmation. Thirdly, in vitro experiments only utilize the HepG2 cell line. The effects of SPP1 on cell proliferation, migration, and invasion should be validated in more HCC cell lines and animal models. Finally, drug sensitivity prediction is based on cell line data from the GDSC database, which may differ from the actual therapeutic effect in clinical patients. It is necessary to validate these results in combination with clinical samples.

Reviewer 3:

1. Figure Legend: Figure 1 legend incorrectly refers to EXOSC10 instead of SPP1, indicating a likely typographical or copy-editing error that should be corrected.

Response: Thank you for your reminder, just like the previous reviewer's comments. I have made further modifications based on your feedback.

2. Overstated Claims: Statements such as “patients with high SPP1 expression are more vulnerable to these medications” are overstated. A more accurate phrasing would be: “predicted drug sensitivity based on transcriptomic modeling.”

Response: Indeed, I have exaggerated the description of the result. I have made further modifications according to your request.

Page 14

Before revise: This suggested that patients with HCC who expressed high levels of SPP1 were more vulnerable to the effects of the previously stated medications, which improved patient outcomes.

After revise: Transcriptomic drug sensitivity analysis predicted that the high-SPP1 subgroup may exhibit increased sensitivity to these medications.

3. Results Section – Potential Pathway of SPP1 Regulation in HCC: The sentence describing cellular component enrichment appears to be duplicated. Specifically, the statement:“In the cellular component, DEGs were mainly concentrated in the collagen-containing extracellular matrix, the apical part of the cell, the apical plasma membrane, and the synaptic membrane, among others (Figure 3B and 3C)” is repeated. The authors should remove the duplicate to improve clarity and avoid redundancy.

Response: Thank you for your reminder. I have deleted the above content.

4. Tumor Immune Microenvironment: The manuscript addresses the tumor immune microenvironment and immune cell infiltration associated with SPP1 expression. However, the analysis primarily relies on computational estimations. It would strengthen the study to clarify whether any crosstalk between stromal cells, resident liver cells, and infiltrating immune cells has been investigated or demonstrated. In particular, discussion or experimental evaluation of interactions among these cellular components within the tumor microenvironment would be valuable. Furthermore, it is unclear whether any co-culture experiments (e.g., tumor cells with immune or stromal cells) were performed to validate the predicted immune interactions. Including such validation or addressing this limitation would enhance the interpretation of the findings.

Response: Thank you for your reminder. I have added this section to the restricted section of my paper.

After revise: Additionally, while we analyzed tumor immune microenvironment and immune cell infiltration related to SPP1 expression using computational estimations, we did not experimentally investigate the interactions between stromal cells, resident liver cells, and infiltrating immune cells. Future research should address these aspects to enhance the interpretability and robustness of our findings.

5. EMT and Metastasis: While the manuscript demonstrates that SPP1 promotes HCC cell proliferation, migration, and invasion, there is no discussion of epithelial-mesenchymal transition (EMT) or metastasis, which are key processes in tumor progression. Including analyses or discussion linking SPP1 to EMT markers and metastatic potential, and referencing any available in vivo or clinical data supporting a role for SPP1 in HCC metastasis, would significantly strengthen the manuscript.

Response: I have placed the relevant discussion content in the discussion section.

Page 16

In addition to promoting proliferation, migration, and invasion, SPP1 may also contribute to hepatocellular carcinoma progression by facilitating epithelial-mesenchymal transition (EMT) and metastatic dissemination. EMT is a crucial biological process in tumor progression, during which epithelial cells lose cell-cell adhesion and acquire mesenchymal characteristics, thereby enhancing motility, invasiveness, and resistance to apoptosis. Previous studies have shown that SPP1 is closely associated with EMT-related phenotypes in multiple malignancies and may regulate the expression of canonical EMT markers, including E-cadherin, N-cadherin, and Vimentin, through pathways such as PI3K/AKT, NF-κB, and TGF-β signaling. In HCC, elevated SPP1 expression has been reported to correlate with more aggressive clinicopathological features and poor prognosis, suggesting a potential role in metastatic progression. In the present study, SPP1 overexpression significantly enhanced the migratory and invasive abilities of HepG2 cells, whereas SPP1 knockdown produced the opposite effect, which is consistent with a pro-EMT and pro-metastatic role of SPP1. Although EMT markers and in vivo metastatic behavior were not directly evaluated in our study, these findings, together with previous reports, suggest that SPP1 may promote HCC aggressiveness at least partly by participating in EMT-related phenotypic plasticity and metastatic potential. Further studies are needed to determine whether SPP1 directly regulates EMT marker expression and metastatic colonization in HCC.
